# Organization and operation of multi particle therapy facilities: the Marburg Ion-Beam Therapy Center, Germany (MIT)

**DOI:** 10.1007/s12553-024-00881-4

**Published:** 2024-05-21

**Authors:** Klemens Zink, Kilian Simon Baumann, Ulrike Theiss, Florentine Subtil, Sonja Lahrmann, Fabian Eberle, Sebastian Adeberg

**Affiliations:** 1 Marburg Ion-Beam Therapy Center (MIT), Department of Radiotherapy and Radiation Oncology, Marburg University Hospital, Albrecht-Kossel-Strasse, Marburg, 35043 Germany; 2 Department of Radiotherapy and Radiooncology, Marburg University Hospital, Baldingerstrasse, Marburg, 35043 Germany; 3 Institute for Medical Physics and Radiation Protection, University of Applied Sciences Giessen, Wiesenstr. 14, Giessen, 35390 Germany

**Keywords:** Particle therapy, Protons, Carbon ions, Synchrotron based facilities

## Abstract

**Purpose:**

The Marburg Ion-Beam Therapy Center (MIT) is one of two particle therapy centers in Germany that enables the treatment of patients with both protons and carbon ions. The facility was build by Siemens Healthineers and is one of only two centers worldwide built by Siemens (Marburg, Germany and Shanghai, China). The present report provides an overview of technical and clinical operations as well as research activities at MIT.

**Methods:**

The MIT was completed in 2011 and uses a synchrotron for accelerating protons and carbon ions up to energies of 250 MeV/u and 430 MeV/u respectively. Three treatment rooms with a fixed horizontal beam-line and one room with a 45 degree beam angle are available.

**Results:**

Since the start of clinical operations in 2015, around 2.500 patients have been treated at MIT, about 40% with carbon ions and 60% with protons. Currently around 400 patients are treated each year. The majority of the patients suffered from benign and malign CNS tumors (around 40%) followed by head and neck tumors (around 23%). MIT is actively involved in clinical studies with its patients. In addition to clinical operations, there is active research at MIT in the fields of radiation biology and medical physics. The focus is on translational research to improve the treatment of H & N carcinomas and lung cancer (NSCLC). Moreover, intensive work is being carried out on the technical implementation of FLASH irradiation for research purposes.

**Conclusion:**

The MIT is one of two centers worldwide that were built by Siemens Healtineers and has been successfully in clinical operation since 2015. The service provided by Siemens is guaranteed until 2030, the future after 2030 is currently under discussion.

## Introduction

Charged particle therapy is regarded as cutting-edge technology in oncology. Worldwide 125 particle therapy centers are in operation and almost 370.000 patients had been treated by the end of 2022 [1]. In more than 90% of these centers, only protons are available for irradiation; only 14 centers worldwide also have the option of using carbon ions. Of these 14 centers, 10 are located in Asia (China, Japan, Taiwan) and four in Europe. Of these four centers, two are located in Germany, the Heidelberg Ion-Beam Therapy center (HIT) located in Heidelberg and the Marburg Ion-Beam Therapy center in Marburg. Both centers are synchrotron-based facilities and act independently from each other.

Radiotherapy with heavy ions^1^ in Germany started already in 1997 with the pilot project at GSI^2^. From 1997 to 2008, GSI has been operating a radiotherapy unit for cancer treatment using carbon ions at its accelerator facility, in collaboration with the Department of Radiotherapy at the Heidelberg University Medical Center, the German Cancer Research Institute (DKFZ) and the Rossendorf Research Center near Dresden. During this time about 430 patients have been treated mostly suffering from tumors in the base of the skull [2–5]. Based on the experience of the pilot project, the University Heidelberg and the University Medical Center Heidelberg started in 2004 to build the first clinical synchrotron based particle therapy center in Germany, the Heidelberg Ion-Beam Therapy Center (HIT). The first patient at HIT was treated in 2009.

Around the same time of the GSI pilot project the company Siemens Healthineers (SHS) decided to develop and build synchrotron based particle therapy centers with the possibility to treat patients with protons and also carbon ions. In a first phase three centers were planned, one in Shanghai (China), one at the University Medical Center Marburg (UKGM) in Marburg (Germany) and one in Kiel (Germany). The centers were built between 2007 and 2012 and each center was designed for a capacity of 2.000 patients per year. During the commissioning of the facilities, it became clear that due to technical limitations, the facilities could only irradiate around 700 - 1000 patients per year. This meant that the facilities could no longer be operated economically and SHS terminated the particle therapy project. Despite these general conditions, the centers in Shanghai started clinical operation in 2014 and the center in Marburg (MIT) in 2015. The facility in Kiel was dismantled. SHS has signed maintenance and service contracts for both facilities and has undertaken to keep them in operation until 2030.

This review article provides an overview of the clinical operation of the facility over the last eight years. In addition, current research projects at MIT are discussed.

## The Marburg Ion-Beam Therapy Center

### Technology at MIT

**Fig. 1:**
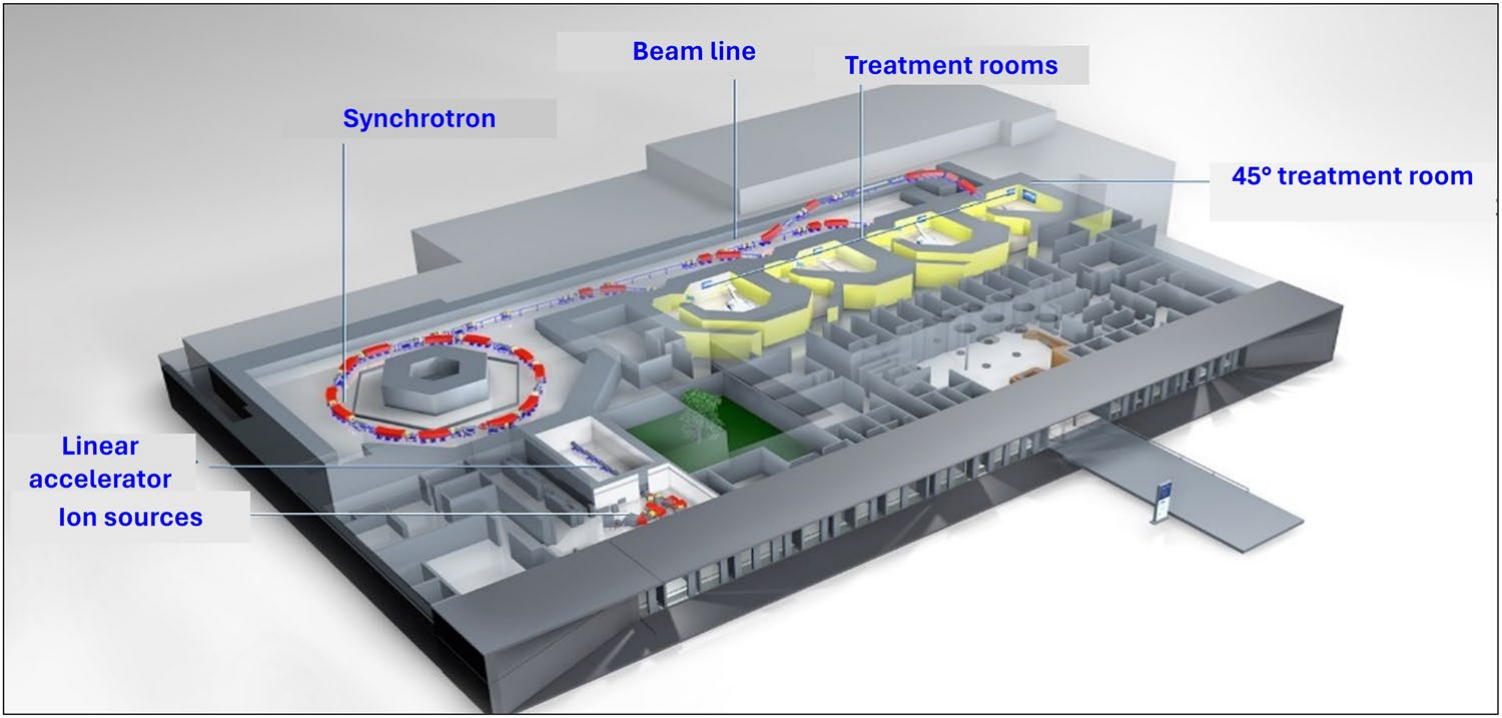
Layout of the Marburg Ion-Beam Therapy Center (MIT). The facility has two ion sources (protons and carbon ions), a linear accelerator for pre-acceleration of protons and carbon ions before injection into the synchrotron. The facility is equipped with four treatment rooms, three rooms have a fixed horizontal beam line, in the fourth room the beam hits the patient at 45°. The synchrotron has a diameter of about 24 m, the dimensions of the whole building are about 110 x 66 m^2^

The synchrotron based accelerator system is able to produce protons in the energy range 48 to 221 MeV/u and carbon ions in the range 86 to 430 MeV/u. These energies lead to penetration depths in the patient of up to around 35 cm. The protons and carbon ions are produced in two different ion sources and injected into a linear accelerator (linac). After passing through the linac, the particles have an energy of around 9 MeV/u and are injected into the synchrotron. Within the synchrotron there is one cavity where the particles are accelerated. The final energy of the particles can be selected in 290 levels. Once the target energy is reached, the particles are extracted and transported into one of the four treatment rooms via the high-energy beamline (see Fig. 1). The extraction is performed via the radiofrequency slow extraction method [6], resulting in spill lengths in the range from milliseconds to several seconds.

All treatment rooms are equipped with the raster scanning beam delivery system [7], allowing a maximum field size of 20 x 20 cm^2^ at the isocenter, which is located 112,6 cm away from the nozzle. Three out of four treatment rooms have a fixed horizontal beam line, in the fourth room the beam hits the patient at 45°. This beam geometry is especially used for the treatment of neuro axis, several head and neck carcinomas and sarkomas of the extremities. A total of around 40% of all patients are treated in this 45°-room.

**Fig. 2:**
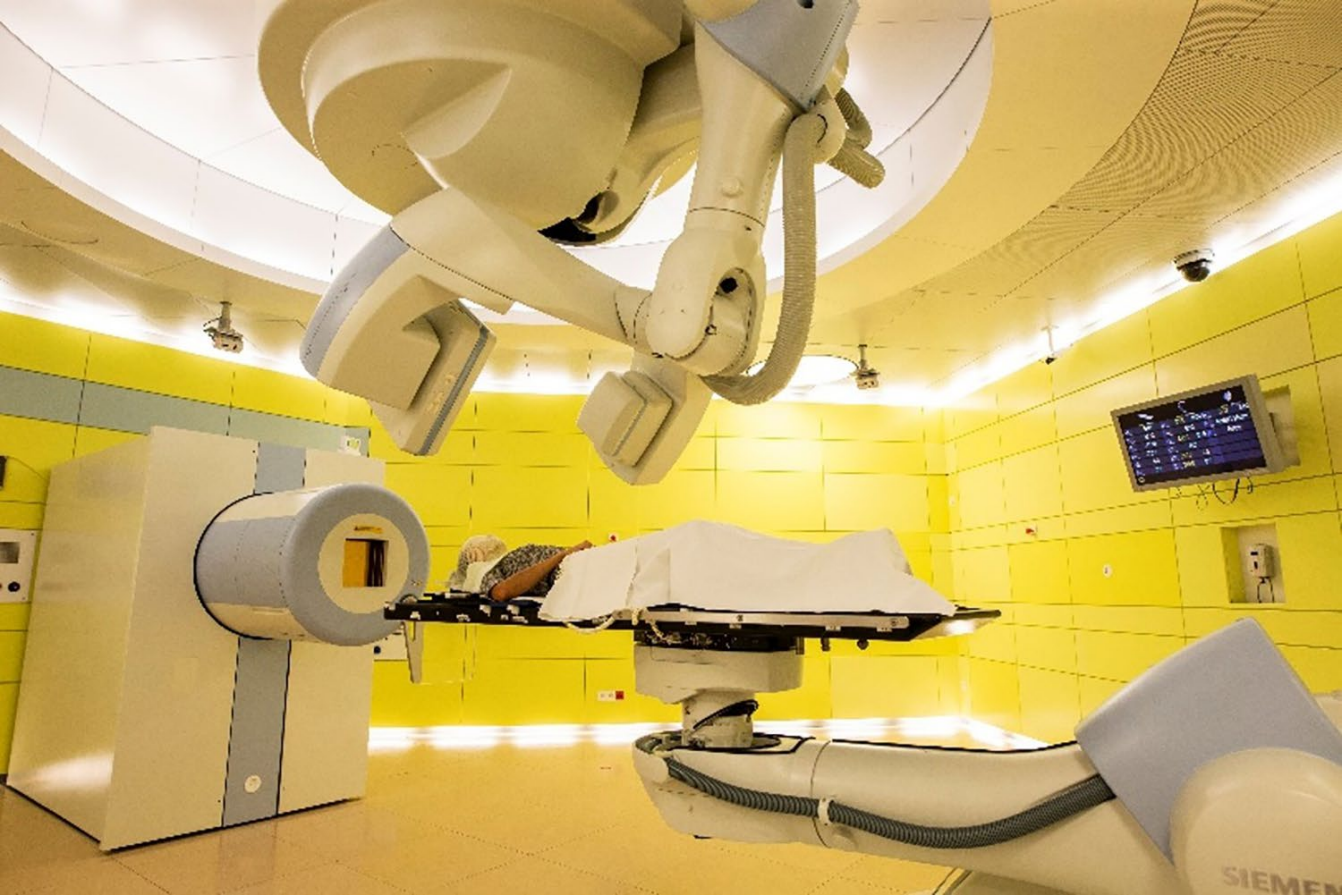
Treatment room at MIT with fixed horizontal beam line. Both the patient table and the X-ray imager are robot-based. In total, the MIT has three identical treatment rooms with horizontal beam line and one room where the beam hits the patient at 45° (Fig. 1)

A C-arm X-ray system is available for positioning patients in all treatment rooms, which is attached to a ceiling-mounted robotic system (Fig. 2). With this system, X-ray images can be taken at any angle, typically, orthogonal images are taken and bone matching of the images with digital reconstructed radiographs from treatment planning is performed.

### Patient treatments

**Fig. 3:**
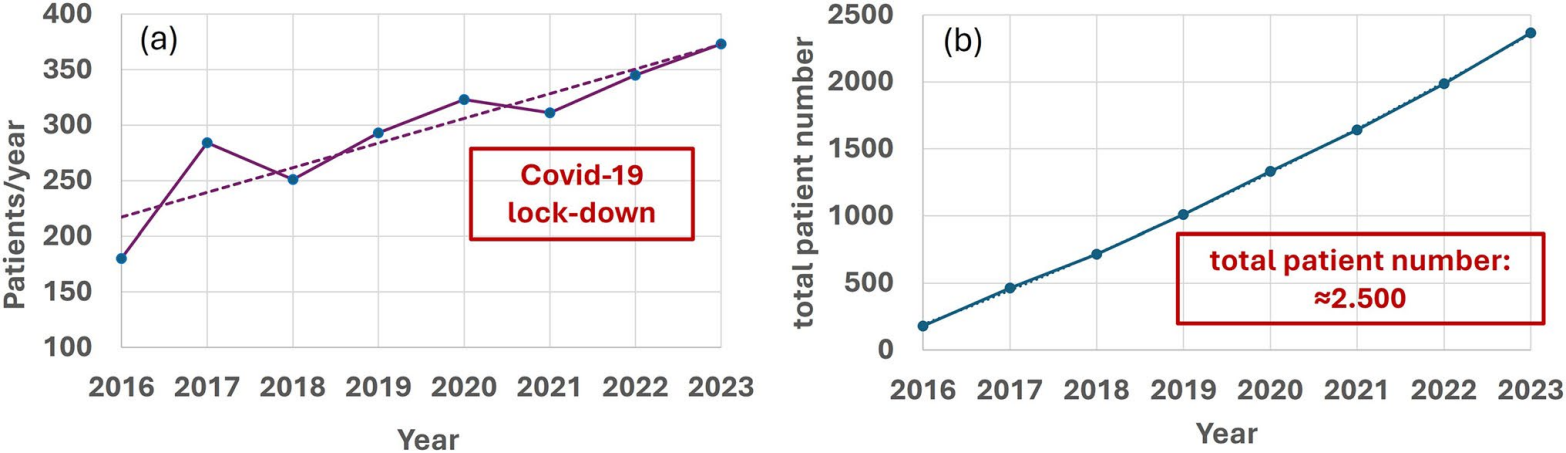
Number of patients treated at MIT. **a** patient number per year. **b** cumulative number of patients

The first patients were treated at the end of 2015. With two exceptions, the number of patients per year has risen continuously since then (see Fig. 3). In the past year 2023, the number of patients irradiated at MIT was 373. A total of around 2,500 patients have been treated since the start in 2015. The exceptions, in which patient numbers did not increase, were 2018 and 2021. The reason for the decline in 2021 was the COVID-19 pandemic. The decrease in 2018 was due to the change of owner/management of MIT that took place at that time. From 2015 to 2018, the MIT was managed by HIT. In 2019, MIT was re-transferred to the University Medical Center Marburg (UKGM).

**Fig. 4:**
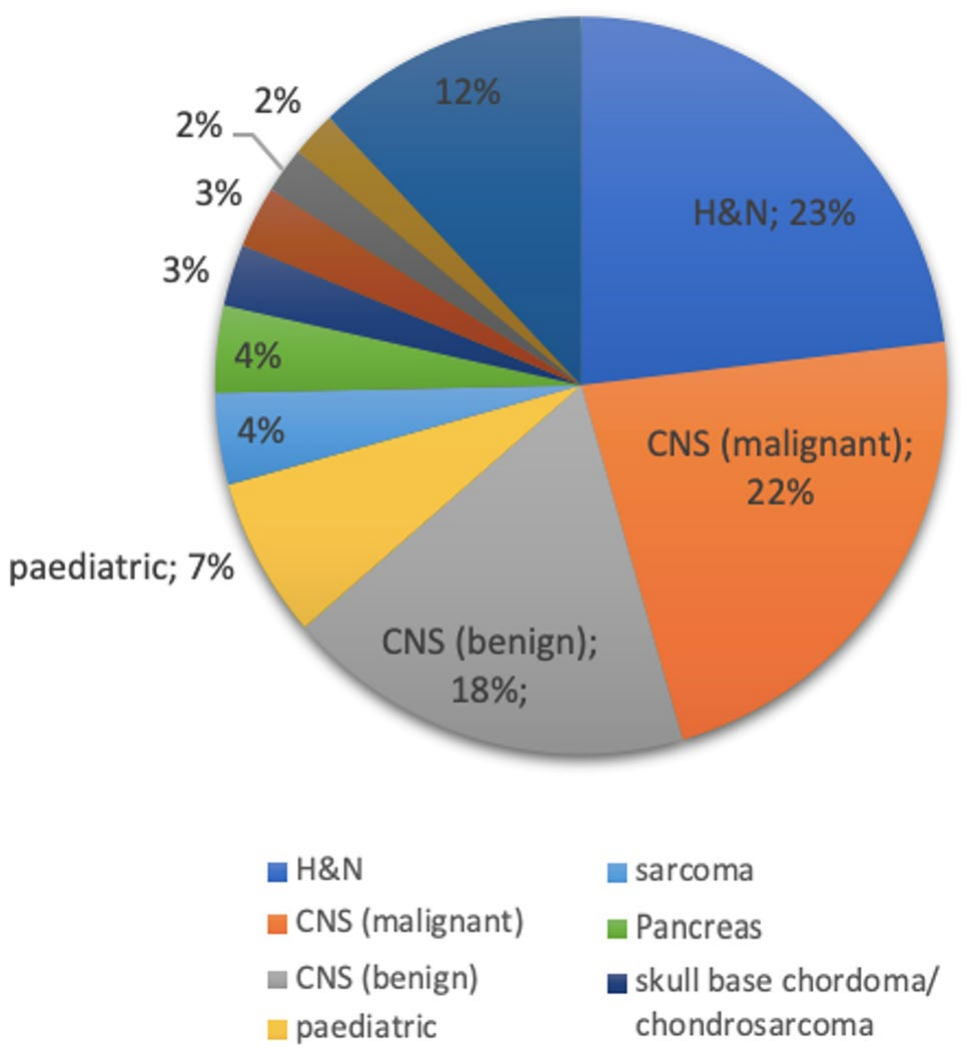
Treated tumor entities and their percentage share of the total collective of all MIT patients. Abbreviations: H & N: head & neck tumors, CNS: central nervous system

Figure 4 shows the tumor entities treated at MIT and their percentage distribution. As can be seen, the largest group with about 40% are patients with tumors in the central nervous system. The second largest group are patients with head and neck tumors. About 7% of all patients at MIT are pediatric tumor patients; the vast majority of them are irradiated under general anesthesia. The ratio of protons to carbon treatment is around 60:40 and the proportion of primary therapies to the total number of treatments is around 65%.

For treatment planning the system Syngo PT (Siemens Healtineers) is used. The system uses a pencil beam algorithm for dose calculation, the relative biological effectiveness (RBE) for carbon ions is calculated with the local effect model [8, 9] in the version LEM I. The reason for using LEM I is to remain comparable to clinical practice at HIT. Generally, $\alpha /\beta -$ratios of 2 Gy are applied in LEM for both tumor and healthy tissues. Exceptions are Pancoast ($\alpha /\beta$ = 10 Gy) and Pancreatic tumors ($\alpha /\beta$ = 5 Gy). In case of protons a constant RBE value of 1.1 is applied.

Patients are currently irradiated in one shift (8 hours), 5 days a week. The number of daily patient treatments is about 35 - 40. The team of about 10 medical physicists works in two shifts. Prior to patient operation, they carry out daily QA procedures in all radiation rooms (safety checks, dosimetry, etc.) and release the rooms for patient operation. All treatment plans are verified by means of measurements before the first patient treatment. The measurements are performed in a water phantom which includes a set of 24 small-volume ion chambers which are fixed in a holder. The holder can be moved in all three directions within the phantom. During verification, the holder is positioned so that most of the chambers are in the high-dose region of the planned dose distribution. For the plan to be used clinically, the mean value of all dose deviations between measurement and treatment plan in the high dose range must not be greater than 5%

The accelerator team consists of around 15 people who operate and monitor the accelerator in 3 shifts, 7 days a week. Maintenance and repair to the accelerator are carried out independently by this team. The machine up-time during the last years was higher than 95%.

## Research at MIT

In addition to clinical research, MIT conducts research in the fields of radiation biology and medical physics. Moreover, MIT supports external groups in their experiments at MIT. The state of Hesse provides funding for beam time at MIT for Hessian research groups to carry out physics and radiobiology experiments. Since 2018, more than 20 scientific projects have been successfully implemented at MIT as part of this funding.

### Clinical research

**Fig. 5:**
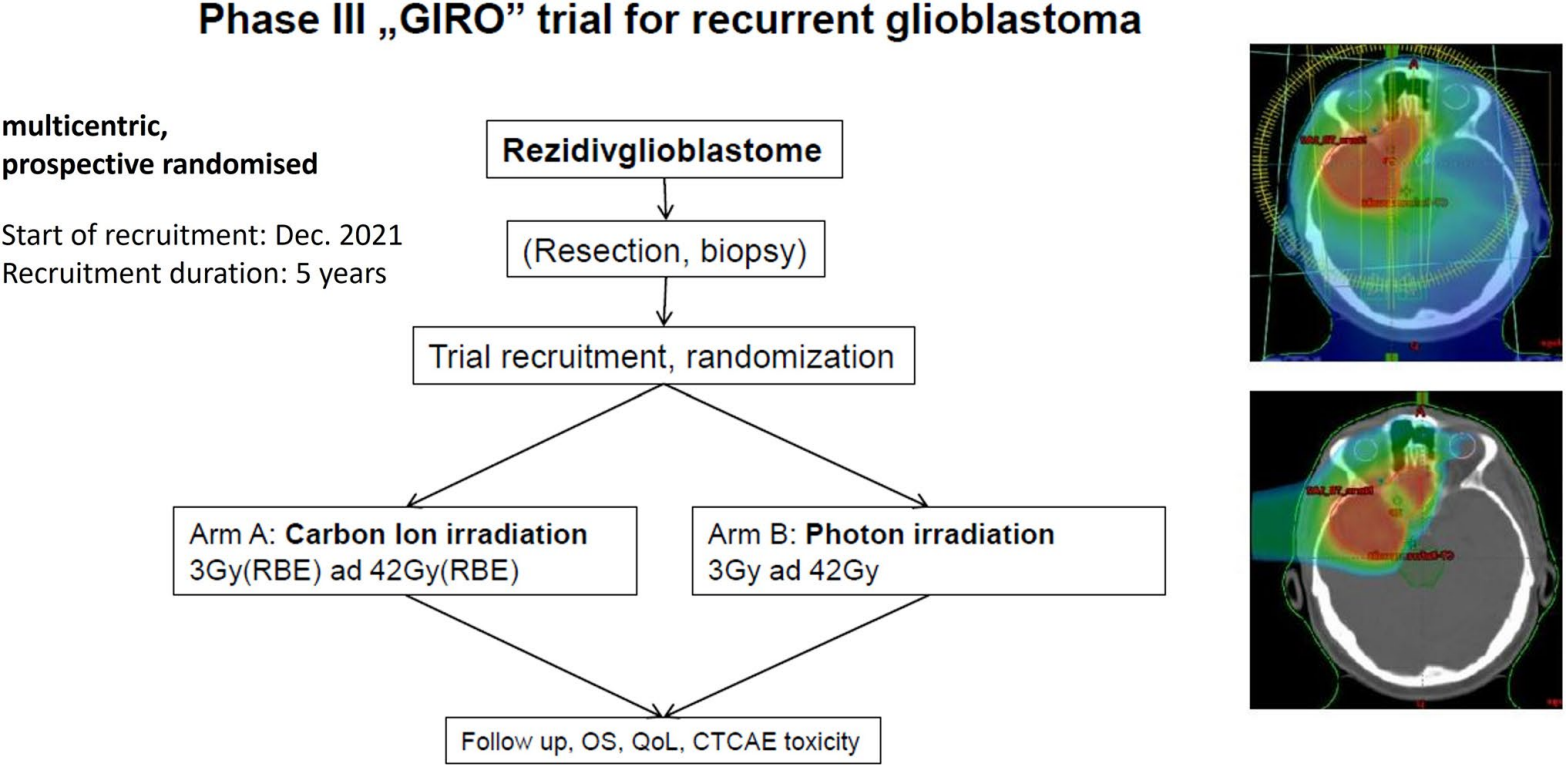
Study protocol for the multicentric, prospective randomised phase III clinical trial *GIRO* for the treatment of recurrent glioblastoma. The recruitment started in 2021

The focus of clinical research at MIT is the treatment of primary and relapsed CNS tumors, especially glioblastomas [10, 11], but also on the treatment of head and neck squamous cell carcinoma (HNSCC), especially in critical areas like the nasal areas or ears [12]. MIT is involved in six clinical studies:

- GliProPh: Randomized phase III trial comparing proton vs. photon radiotherapy for patients with WHO grade II-III gliomas;
- SIOP EPENDYMOMA 2: Randomized therapy optimization registry study for the treatment of children, adolescents and young adults with ependymoma [13];
- GIRO: Randomized phase III trial comparing carbon ion vs. photon radiotherapy for patients with recurrent glioblastoma (Fig. 5);
- GRIPS: Randomized phase II trial comparing proton vs. photon radiotherapy for patients with glioblastoma;
- PAROS: Randomized phase III trial - Prostate cancer irradiation with alternative radioncological approaches [14]
- INSPIRE: Prospective, organ-specific registry study

### Radiation biology

**Fig. 6:**
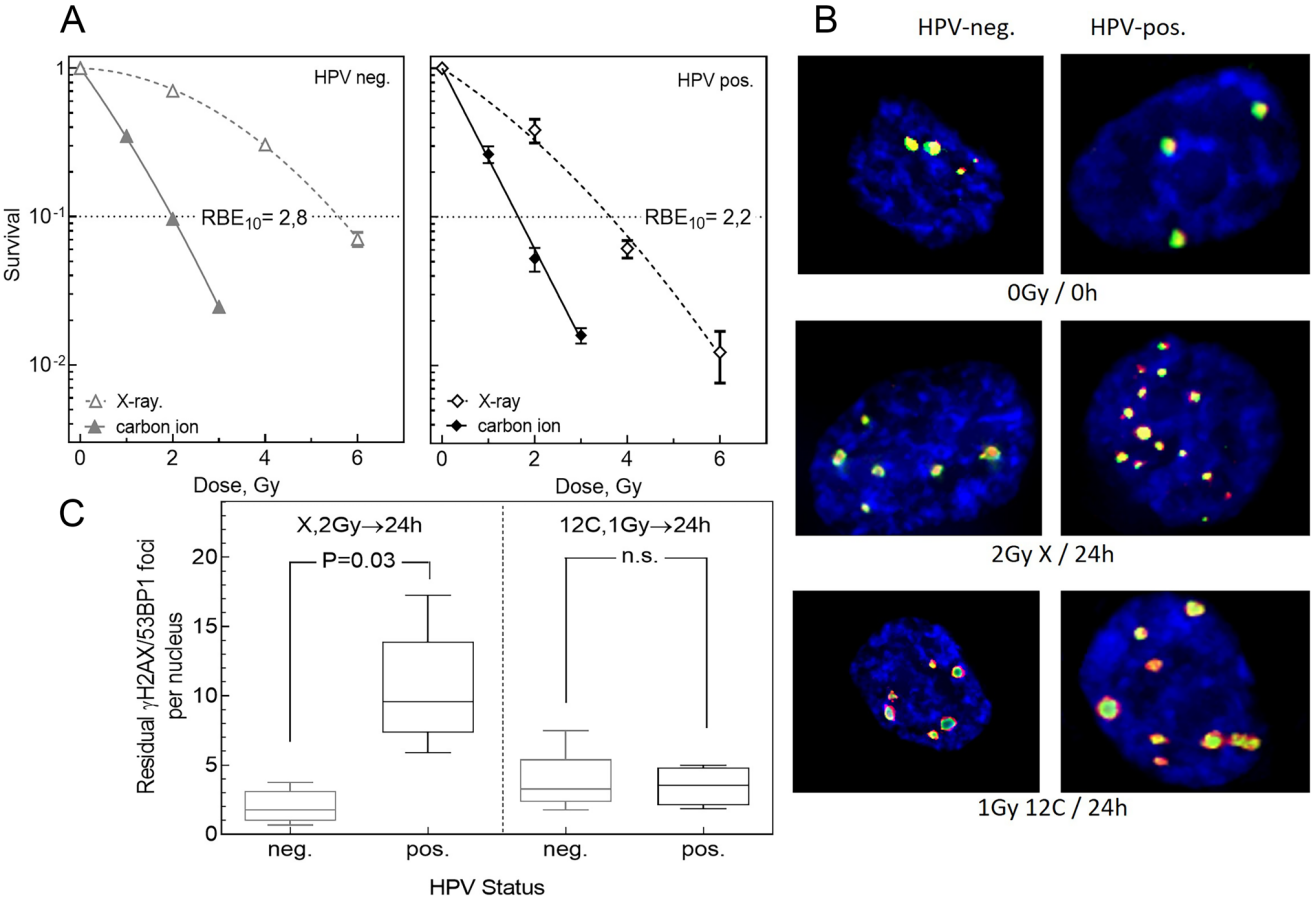
DNA damage and survival of HPV negative (neg.) and positive (pos.) cells after X-ray and carbon ion irradiation. Experiments were performed with five HPV negative (FaDu, UM-SCC-3, UM-SCC-6, UM-SCC-11b, UT-SCC-33) and five HPV positive (UD-SCC-2, UM-SCC-47, UM-SCC-104, 93VU147T, UPCI:SCC-154) HNSCC cell lines [15]. **A** Survival was detected via clonogenic assay for one HPV pos. and one HPV neg. HNSCC cell line. Cells were irradiated with various doses of X-ray or carbon ion irradiation and the relative biological effect (RBE) was calculated at 10% cell survival. **B** Double strand breaks were detected 24 h after irradiation via co-staining of $\gamma$H2AX/53BP1. **C** Double strand breaks were measured for five HPV pos. and five HPV neg. HNSCC cell lines after irradiation with 2 Gy X-rays or 1 Gy carbon ions. Significance level $p<$ 0.05, n.s. not significant

Radiotherapy is an essential part of multidisciplinary treatment of head and neck squamous cell carcinoma (HNSCC). Whereas HPV-positive HNSCC benefit from the conventional treatment options, for individuals with HPV-negative HNSCC recurrence is a common event, despite intense therapeutic approaches. Irradiation with carbon ions is a relevant alternative treatment option. Due to its increased relative biological effectiveness (RBE) an increase in cell killing can be achieved and the high spatial accuracy in energy deposition allows for reduced toxicities in normal tissues. Preclinical studies demonstrate an improved cell killing for HPV-positive and HPV-negative HNSCC cells (Fig. 6a) [15]. However, the RBE for HPV-positive cells is lower with 2.2 compared to HPV-negative cells with 2.8 (Fig. 6b, c). One possible explanation for this result could be that the higher RBE of the HPV-negative cell lines is a result of resistance of this cell lines against photons and the fact that both cell lines show similar radio-sensitivity against carbon ions. Thus, biological differences are only of minor importance for the response to carbon ions and therefore lower tumor dose leading also to lower doses in the adjacent normal tissue are sufficient when using carbon ions. These results have to be considered when clinical protocols are established.

Currently carbon ion irradiation is used for head and neck tumors located in critical areas (f.i. nasal cavity, ear) [12] or in second-line treatment [16–18] with good clinical results that argue for a broader clinical practice. Moreover, the multifaceted mechanisms of therapy resistance, exhibited by HNSCC tumors, such as inflammatory and immune-modulating cytokine and chemokine signaling or overstimulation of the PI3K/AKT/TOR signaling pathway are alleviated for carbon ion irradiation [19, 20]. In this context, [15] is a valuable source to plan further clinical studies.

Non-small-cell lung carcinoma (NSCLC) is another tumor entity that can strongly benefit from carbon ion irradiation. With a very poor prognosis, it is the most common cause of cancer death in Germany. Therapy resistance is high in NSCLC, and the current therapies fail for most individuals. Photon irradiation triggers HIF-1 and AKT/mTOR signaling pathways in this tumor. Moreover, the induction of VEGF can trigger angiogenesis and metastasis. In vitro and in vivo studies of NSCLC treated with carbon ion irradiation show a clear advantage for this radiation quality, as it does not enhance any of these pathways that cause treatment resistance [21–23]. Clinical data supports these encouraging results from experimental studies. Observational studies show that the 5-year overall survival can be increased from 20% for photon, to 40% for proton and up to 42% for carbon ion treatment [24]. Other clinical studies present even more impressive numbers with 58.7% - 70% overall survival, but observation times are lower with only 2 years [25, 26]. Moreover, normal tissue toxicities were decreased.

To gather more clinical evidence, a corresponding study is currently executed at MIT (PARTITUR). A network of experts in the field of Medical Physics, Radiobiology and Lung Cancer research is interacting to optimize the particle irradiation techniques for the treatment of lung cancer.

### Medical physics

The raster scan method allows high-precision irradiation of tumors with protons or heavy ions. However, if the tumor moves, e.g. due to the patient’s breathing, interplay effects result in inhomogeneous dose distributions in the tumor and the surrounding healthy tissue, which are typically not tolerable. In order to minimize these undesirable dose inhomogeneities, various methods such as gating [27], re-scanning [28] and 4D-planning and treatment methods [29–31] have been developed. The disadvantage of these methods, however, is the associated increase in irradiation times for the patient.

**Fig. 7:**
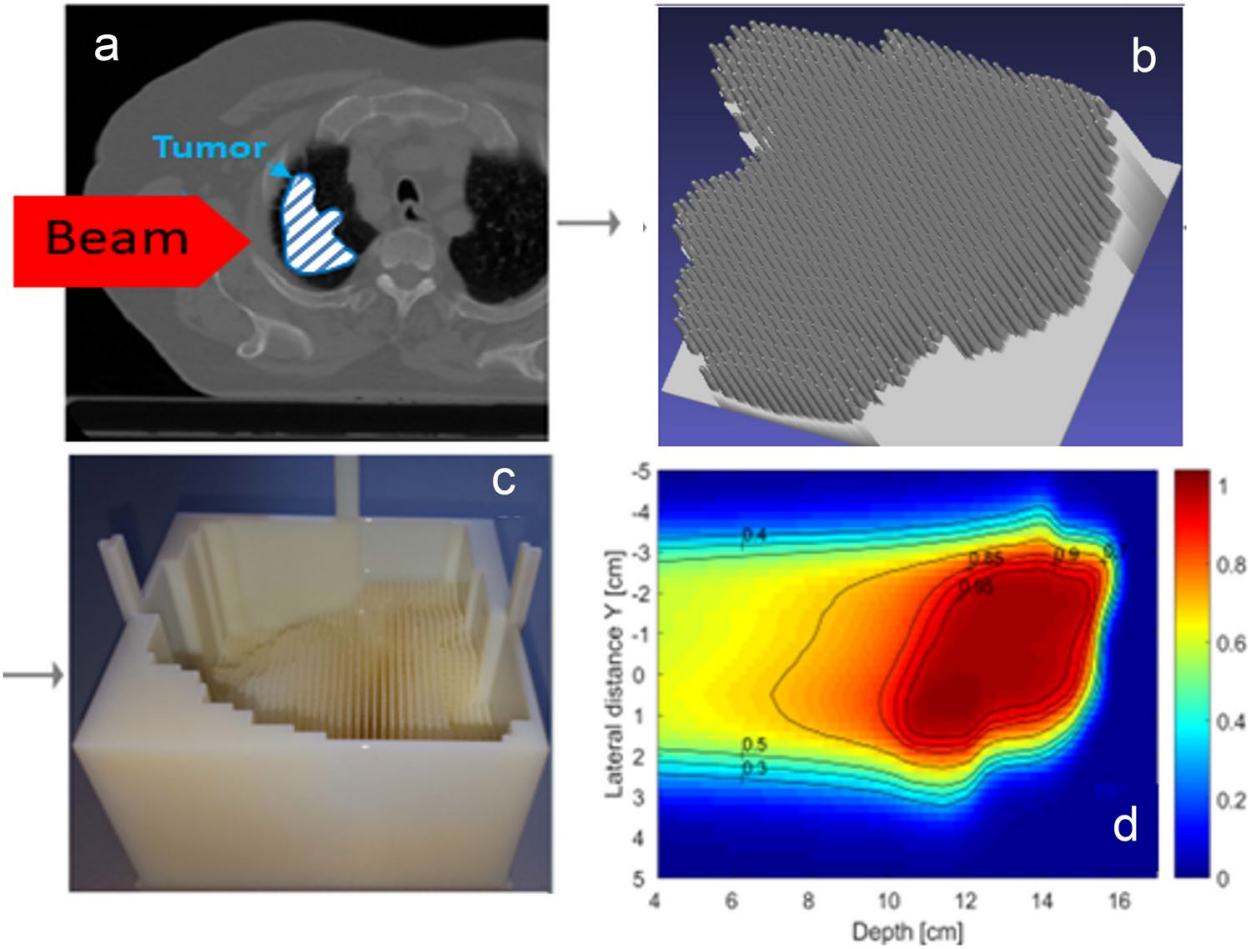
3D range modulator for fast patient treatments in particle therapy. When a monoenergetic ion beam passes through the range modulator, which consists of a large number of fine pins (base area typically 3 x 3 mm^2^), there are locally different energy losses of the ions, resulting in the formation of differently extended Bragg peaks (SOBP’s) with different maximum penetration depths in the tissue. By optimizing the geometry of the modulator to the shape of the tumor, a homogeneous dose coverage in the planned target volume is achieved. **a** CT of the patient with tumor; **b** virtual 3D model of the 3D range modulator; **c** printed 3D range modulator; **d** measured dose distribution behind the range modulator in a water phantom. The modulator was scanned once with monoenergetic protons with an energy of 150 MeV/u [34]

To speed-up delivery time, the 3D range modulator was developed at MIT together with GSI. It is a passive component that is introduced into the beam. The locally varying energy modulation of a monoenergetic particle beam as it passes through the modulator enables the production of an extended spread-out Bragg peak (SOBP) at each individual point within the treatment field, whereby the width and position of the SOBP at each point of the treatment field can be adapted to the requirements of the target volume. That means that even complex target volumes can be irradiated with a conformal dose distribution using a single monoenergetic particle radiation field. As a result, the irradiation time can be reduced to a few seconds or even milliseconds, thus preventing respiratory-related tumor movements by breath holding. The procedure is show in Fig. 7. Further details can be found in [32–34].

3D-range modulators are also of great importance for so-called FLASH treatments, where the patient is treated with very high dose rates ($D>$ 40 Gy/s). Both radio-biological and animal experiments show that with FLASH irradiation the radiation effect on the tumor is comparable to the effect with conventional dose rates ($D \approx$ 0.1 Gy/s), but the effect on normal tissue is significantly reduced [35–38]. For many years oxygen depletion had been discussed as a possible mechanism for reduction of the healthy tissue damage after exposure to ultra-high dose rates. However, the mechanism underlying the FLASH effect remains to be elucidated.

In order to perform experiments with ultra-high dose rates at MIT with both protons and carbon ions, a fixed parametrization of the beam extraction system installed in the synchrotron has been implemented to extract as many particles as possible in a fixed time. This extraction time has been set to 150 ms for carbon ions and 100 ms for protons for optimal extraction efficiency. For carbon ions, up to 8.3 $10^8$ particles can be extracted resulting in a dose rate of 230 Gy/s at the entrance channel region when a spot of 8.6 mm in width is being applied. For protons, up to 1.8 $10^{10}$ particles can be extracted resulting at a dose rate of 275 Gy/s. Using these setting, experiments have been performed to investigate the change in the production of reactive oxygen species, such as hydrogen peroxide (H_2_O_2_), under FLASH conditions compared to conventional dose rates. It has been shown that ultra-high dose rates lead to a reduced production of (H_2_O_2_) [39]. Interestingly and in contrast to the experimental investigation of the production of (H_2_O_2_), Monte Carlo simulations performed at our working group show an increase of (H_2_O_2_) under FLASH irradiation [40–42]. This discrepancy is subject of the ongoing research at our working group.

Last but not least the medical physics group at MIT is working on improving the dosimetry of protons and carbon ions. Recently, the IAEA TRS-398 Code of Practice (CoP) [43] was updated. For this update, the Monte Carlo codes FLUKA [44–46] and Geant4 [47] have been used in our working group to derive beam quality correction factors $k_Q$ for various cylindrical and plane-parallel air-filled ionization chambers in clinical proton beams [48–53]. These results have been incorporated in the update of $k_Q$ factors of the new TRS-398 CoP and have helped to reduce the uncertainty of tabulated $k_Q$ factors down to 1.4%.

## Discussion and conclusion

The synchroton-based facility MIT is one of two particle therapy centers in Germany that enables the treatment of patients with both protons and carbon ions. Since the start of clinical operations in 2015, around 2.500 patients have been treated. With around 400 patients currently being treated each year, the facility’s capacity is around 70-75%. The task in the coming years will be to increase the number of patients to around 500 in order to justify the high level of investment required during the next years.

Around 15 years have passed since the original planning of the facility in Marburg and during this time there has been rapid development in the field of conventional photon radiotherapy. This affects areas of treatment planning (4D-planning, robust planning, AI-based contouring, etc) but also imaging (3D/4D imaging in the treatment room) and systems to support patient positioning (surface guidance). These innovations have also to be established in particle therapy at MIT. In concrete terms, this means that in the near future the current Syngo PT treatment planning system (TPS) has to be replaced by a modern TPS including the options for Monte Carlo-based dose calculation, robust planning, different models for RBE calculations (LEM and microdosimetric kinetic model (MKM)) etc. Inside the treatment rooms, CT’s has to be installed for 3D imaging to improve the accuracy of patient positioning especially in cases of necessary soft tissue matching and also to start whith adaptive treatment workflows.

In addition to the clinical applications of particle therapy, research activities at MIT will also be further expanded in the future. In the area of clinical research, the aim is to participate in further clinical studies and establish new study protocols.

The acquisition of extensive research funding (PARTITUR, see Acknowledgements) will enable the focus on "Non small cell lung cancer (NSCLC) and particle therapy" to be expanded at MIT over the next years. Radiobiology will focus on the questions of increased inactivation of tumor cells by means of carbon ion therapy. This requires a detailed characterization of damage generation after carbon ion irradiation as well as a comprehensive analysis of the repair processes involved. Based on this, the possibilities of targeted radiation sensitization after carbon treatments will be investigated, with a particular focus on the inhibition of certain signalling pathways within the tumour cell. In addition, in order to achieve a further enhancement of the radiation effect in the NSCLC cells, the prerequisites for an optimal combination of carbon treatment with targeted immunotherapy will also be investigated.

In the field of medical physics, the biophysical model (LEM) essential for the clinical use of carbon ions for the prediction of RBE are to be further developed and relevant basic data are to be compiled and validated with the help of radio-biological data. These investigations are supported by Monte Carlo simulations on micro- and nanometer scales. In addition, important technological as well as physical and dosimetric prerequisites for the irradiation of moving lung tumors with carbon ions are to be created. The focus here is on the further development of the 3D range modulator (Fig. 7) in order to shorten irradiation times to the range of seconds and thus eliminate the problem of tumor movement during irradiation.

The company Siemens Healthineers will continue to look after the two particle therapy centers it has built in Shanghai and Marburg until 2030. The future of MIT after this date is currently under discussion. As the whole facility including the accelerator is a medical device according to the EU Medical Device Directive (MDD), the continued operation must be carried out in accordance with the MDD rules. There are several options doing this, one option could be to operate the facility as a so-called In-house production in accordance with MDD. What this means in detail is currently being examined.

## Footnotes

1 According to the ICRU [54], carbon ions do not count as heavy ions, but all ionic species up to atomic number Z = 10 count as light ions, which is why one should actually speak of light ion therapy. In the literature, however, the term heavy ion therapy has become established in the case of therapy with carbon ions, which is why the term is also used here.

2 GSI Helmholtzzentrum für Schwerionenforschung Darmstadt, Germany
